# Water Quality Metrics of Fishponds During the Cold Season, with a Focus on the Potential Risk of Metals and Microplastics

**DOI:** 10.3390/toxics14050403

**Published:** 2026-05-08

**Authors:** Marinela Mirica Gancea, Cristiana Radulescu, Andreea Laura Banica, Ioana Daniela Dulama, Raluca Maria Stirbescu, Ioan Alin Bucurica, Mioara Costache, Mariana Cristina Arcade

**Affiliations:** 1 Nucet Research and Development Institute for Fish Farming, Academy of Agricultural and Forestry Sciences “Gheorghe Ionescu Şişeşti”, 137335 Nucet, Romania; marinella1968@yahoo.com (M.M.G.); mioaracostache48@yahoo.com (M.C.); arcademarianacristina@gmail.com (M.C.A.); 2 Doctoral School of Chemical Engineering and Biotechnology, National University of Science and Technology Politehnica Bucharest, 060042 Bucharest, Romania; banica.andreea@icstm.ro; 3 Faculty of Sciences and Arts, Valahia University of Targoviste, 130004 Targoviste, Romania; 4 Academy of Romanian Scientists, 050044 Bucharest, Romania; 5 Institute of Multidisciplinary Research for Science and Technology, Valahia University of Targoviste, 130004 Targoviste, Romania; stirbescu.raluca@icstm.ro (R.M.S.); bucurica_alin@icstm.ro (I.A.B.); 6 Doctoral School Plant and Animal Resources Engineering and Management, University of Agronomic Sciences and Veterinary Medicine of Bucharest, 011464 Bucharest, Romania

**Keywords:** fishpond, potentially toxic metal, microplastics, risk, statistical approach

## Abstract

Aquaculture in ponds supplied by streams or rivers requires careful evaluation of key physicochemical parameters and potential pollution threats, particularly metals and microplastics. To address these challenges, this research aims to monitor daily climatic and physicochemical parameters and quantify potentially toxic metals and microplastics in the water of 19 fishponds in the SCDP Nucet, Romania, over one winter season (i.e., December 2024 to February 2025). During this season, unique hydrochemical conditions arise, such as lower temperatures, reduced light, and decreased activity, which can affect the ecological balance and fish health. Accordingly, a total of 4650 samples were collected and analyzed in terms of physicochemical parameters (i.e., alkalinity, bicarbonate, calcium ions, magnesium ions, Ca^2+^/Mg^2+^ ratio, organic matter, nitrates, nitrites, phosphates, ammonium, total hardness, resistivity, dissolved oxygen, conductivity, salinity, turbidity, free and total chlorine), metals, and microplastics. Statistical analysis revealed the influence of winter weather on water quality, highlighting links between air and water temperatures and physicochemical parameters. Furthermore, water analyses revealed notable levels of microplastics, including fibers and fragments of various colors, shapes, and sizes. Polypropylene, polyethene, and nylon were the most prevalent. While appreciable quantities of blue, green, black, and yellow fibers were found in size ranges (0.09–0.3 mm), irregular yellow fragments or translucent particles were found in sizes less than 0.5 mm. Metal (i.e., Cr, Fe, Ni, Co, Cu, Zn, Cd, and Pb) concentrations do not exceed the standard values set by national and European regulations. However, it is worth noting that microplastics can amplify or mitigate metal toxicity. The results emphasize the importance of integrated monitoring of physicochemical parameters and emerging pollutants during the cold season, thereby improving understanding of the chemical processes governing water quality in fishponds, providing scientific support for future environmental risk assessment, and promoting innovative, adaptive technologies.

## 1. Introduction

Global warming is causing significant changes in the location, timing, and frequency of extreme weather events, and higher temperatures are expected to shift the geographical distribution of climate zones [1,2,3,4,5,6]. The variability of the seasonal average air temperature index is reflected in increased frequency and intensity of extreme heat events and cold waves, especially during winter and early spring [7,8]. The thermal range of aquatic ecosystems in the temperate areas falls within the range (−30 °C, +40 °C), which determines a water temperature between +2 °C and +30 °C [9,10,11]. In Romania’s specific climatic conditions, the average water temperature in fishponds during winter is typically between 2 °C and 5 °C. During the cold season, the metabolic activity of fish decreases to a state of physiological rest, like hibernation. With the increase in water temperature during the transition to spring, metabolic processes gradually reactivate and intensify, with an approximate doubling for each 10 °C increase in temperature [12,13,14]. Winter temperature fluctuations can disrupt metabolic processes and reproductive cycles, thereby directly affecting species that synchronize their reproduction with seasonal temperatures [15,16,17]. Water quality parameters vary seasonally [18,19], and typically, the water sources for fishponds (i.e., rivers and streams) carry contaminants from effluent discharges, which can alter water quality [20,21]. Atmospheric pollutants present during torrential rains, together with untreated wastewater, are considered the most critical pathways for the entry of emerging contaminants (e.g., potential toxic metals, pesticides, toxic organic compounds such as polycyclic aromatic hydrocarbons, volatile organic compounds, dioxins, etc.) into rivers, lakes, and ponds [22,23].

Fishponds are complex aquatic ecosystems in which the interactions among physicochemical and biological factors determine the health and productivity of fish species [24]. Parameters such as temperature, pH, dissolved oxygen, conductivity, and the chemical composition of water play an essential role in regulating metabolic processes and maintaining ecological balance [25,26]. Although the physicochemical parameters of water have been intensively studied during warm seasons, their dynamics during cold periods, especially under ice cover, which is correlated with reduced fish metabolism, remain insufficiently documented, particularly in the specific conditions of fishponds [27]. The cold or wintering season for fish species is recognized as a critical period in freshwater aquaculture systems due to low temperatures, reduced biological activity, and ice formation [28,29,30,31]. These conditions limit gas exchange and favor the accumulation of toxic substances, such as ammonia and nitrites, which can induce physiological stress and metabolic imbalances in fish. Emerging contaminants are chemical compounds that have recently been regulated and for which there are concerns about their environmental impacts and, implicitly, on human health, through the interactions and mechanisms they generate at the ecosystem level [32,33]. In recent years, emerging contaminants, such as metals and microplastics (MPs), have posed an additional threat to fish ecosystems; microplastics persist in water and sediments and can function as adsorption surfaces for metals and other pollutants, increasing the risk of bioaccumulation and ecotoxicological effects [34,35,36,37]. Studies on metal pollution in freshwater aquaculture conducted near urban or agricultural areas are fewer than those focused on marine and oceanic areas [38,39]. Metal pollution of aquaculture water can negatively affect the health of fish species, as these metals tend to bioaccumulate, and their compounds are deposited in various organs and tissues more rapidly than they are metabolized [40,41].

This research investigated how hydrochemical parameters evolve in fishponds during the cold season of 2024–2025, a critical period characterized by reduced biological activity, ice forming on pond surfaces, and limited water exchange. Additionally, elements such as potassium, sodium, and calcium are important to fish because they play essential roles in biological pathways. Other metal(loid)s, such as selenium, lead, chromium, nickel, mercury, and cadmium, are not needed for metabolic processes and can be harmful to fish at extremely low concentrations, as they induce oxidative stress by generating free radicals and reactive oxygen species within cells. Therefore, determining the concentrations of metals as oxidative stressors in fishponds is a crucial step in creating the best environment for implementing new biotechnologies with reduced environmental impact. Another goal of this study was to identify potential sources of metals, including agricultural activities (e.g., aquafeeds, pesticides, and fertilizers), industrial production, and natural resources. Finally, quantifying potentially toxic metals and microplastics, can provide a scientific basis for minimizing their input and accumulation in freshwater aquaculture ponds, as well as offering insights into preventing and controlling ecological risks. This study employed several analytical methods to achieve its purpose. These methods ranged from electrochemical measurements of pH, conductivity, total dissolved solids, salinity, redox potential, chloride, and turbidity to inductively coupled plasma mass spectrometry for metal concentrations and micro-FTIR mapping to characterize smaller microplastics. Finally, statistical analysis was used to assess the health risks posed by contaminants in the study area, accounting for the potential ecological risks associated with pollution sources.

The novelty of this study lies in its purpose: to evaluate the seasonal dynamics of hydrochemical parameters (i.e., alkalinity, bicarbonate, calcium ions, magnesium ions, Ca^2+^/Mg^2+^ ratio, organic matter, nitrates, nitrites, phosphates, ammonium, total hardness, resistivity, dissolved oxygen, conductivity, salinity, turbidity, as well as, free and total chlorine) and metal content (i.e., Cr, Fe, Ni, Co, Cu, Zn, Cd, and Pb) in fishponds during the cold season, thereby contributing to the development of effective, sustainable management strategies and the reduction in ecological risks in freshwater aquaculture.

## 2. Materials and Methods

### 2.1. Site Description

**Figure 1:**
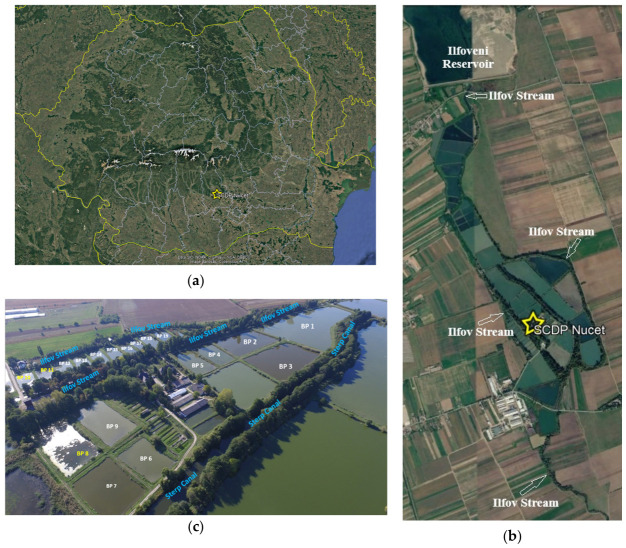
Site description—SCDP Nucet: (**a**) SCDP Nucet in Romania map; (**b**) SCDP Nucet with Ilfov Stream—the supply and the discharge water body; (**c**) the monitored fishponds during the cold season. Note: All images are processed from Google Earth Pro.

The present study was carried out between November 2024 and March 2025 at the Nucet Fish Farming Research and Development Station (well known in Romania as SCDP Nucet, Figure 1a). The study focused on monitoring the main physicochemical parameters in fishponds and the water supply source (WS) during the cold season. The institution is in the southwestern part of Dâmbovița County (in the southeastern part of Romania, Figure 1b) and has ~90 ponds covering a total surface area of 160 hectares. The fishponds are strategically located along the main riverbed of the Ilfov Stream, in a downstream hydrographic sector of the Ilfoveni Reservoir. The water supply system is gravity-fed and energy-efficient, constantly capturing the necessary flow from this natural watercourse. The water used in technological aquaculture processes is subsequently discharged back into the same stream, thereby upholding the semi-closed-circuit principle (Figure 1c). This setup enables quantitative and qualitative control of the water supply and the synergistic integration of fish farming activities into an aquatic ecosystem with lentic characteristics that are favorable to the growth of cyprinids in polyculture.

The studied ponds were stocked with fish species under controlled conditions of growth and reproduction at the SCDP Nucet for wintering. These ponds are typical man-made (anthropogenic) freshwater systems in which biological activity (living organisms such as fish and plants) interacts with physicochemical processes (physical and chemical characteristics of the water, such as temperature, pH, and dissolved oxygen). The ponds were built to meet technological requirements for dimensions (surface area and depth), shape, and location (Figure 1c). Each pond has its own water supply and discharge facilities. The Ilfov Stream supplies water through two branches and the Sterp Canal diversion, with flow regulated by two weirs (structures that control water movement). During this period, water flow was 2–4 L/s/ha, considered sufficient for fish wintering (enough water to support fish survival).

The ponds intended for the wintering of fish stock have an area between 2000 and 5000 m^2^ and a depth in the range of 1.5–2.4 m. The fish material was stocked at the beginning of November 2024, based on its age and size; the density in the pond was 2300–2500 kg/ha, considered adequate for maintaining the fish’s physiological state at low temperatures and reduced metabolic activity. This density prevents overcrowding, ensures a sufficient concentration of dissolved oxygen, and limits the accumulation of toxic metabolites. According to recommendations in the literature [42,43], the optimal range for cyprinid species in semi-intensive farming systems is between 2000 and 3000 kg/ha, depending on hydrobiological parameters and the basin’s self-purification capacity.

### 2.2. Sampling and Sample Preparation

Water sampling was conducted in accordance with the Water Sampling Protocol, which is based on ISO 5667-3:2024 [44]. This protocol concerns water quality, sample preservation, and handling. The procedure is critical for assessing aquaculture water quality. It ensures that physicochemical parameters are represented accurately. Out of approximately 90 ponds in SCDP Nucet, 19 were selected for monitoring. The Ilfov Stream water supply source was also included, bringing the total to 20 sampling areas (Table 1). For each pond, water quality data were collected daily at several points over the three winter months (December 2024 to February 2025). This period corresponds to the fish species’ wintering period. Sampling points were placed along the pond edges and in the corners. Specifically, for each monitored pond, 8 water samples were collected per day. This equals 240 samples/pond/month and 720 samples/pond over the winter period. Since 19 ponds were selected, the total number of analyzed samples was 4560. Additionally, 90 water samples were collected from the water supply source over 3 months (one sample per day). In total, 4650 samples were collected from the 20 areas in SCDP Nucet. These samples were analyzed for physicochemical parameters and trace metals relevant to water quality and fish health during the 2024–2025 winter. For physicochemical analysis, one representative water sample was prepared from each pond daily. All sampling, preservation, and analysis procedures followed the Standard Method for the Examination of Water [44,45].

Water samples were collected from a depth of approximately 30–40 cm below the surface, using clean glass containers, previously rinsed with water from the sampling site. After sampling, the glass containers were labelled with the date, time, location, and sample code. The containers were temporarily stored in portable refrigerated boxes at approximately 4 °C. Samples were transported to the laboratory on the same day. During transport, conditions were maintained at a lower temperature, and samples were protected from light and mechanical shocks. Laboratory analyses were performed within 24 h of sampling [44]. Particular attention was paid to avoiding sample contamination and exposure to atmospheric air during sensitive determinations, such as dissolved oxygen, nitrate, and phosphate concentrations. Before analysis, the samples were temporarily stored under controlled conditions at low temperatures and away from light, as required by each analysis. This sampling protocol maintained the physicochemical integrity of the samples until laboratory analysis.

The two categories of monitored parameters were: essential physical parameters (i.e., turbidity, and resistivity) and chemical parameters (dissolved oxygen, pH, salinity, conductivity, nitrites, chlorides, alkalinity, bicarbonate, and hardness). In parallel, pH and dissolved oxygen levels were measured daily at 08:00, 12:00, and 16:00 using a portable device to capture the diurnal variations in these critical parameters. The multiparameter WTW inoLab Multi 9430-IDS (WTW1FD47K, Fisher Scientific, Loughborough, Leicestershire, UK) was used to assess pH, dissolved oxygen, conductivity, resistivity, and salinity. Free and total chlorine levels were measured with the HACH Pocket Colorimeter II filter photometer (HACH, Loveland, CO, USA), and turbidity was measured with the Mi 415 Turbidimeter (Milwaukee Instruments, Rocky Mount, NC, USA).

Volumetric analytical methods are essential for evaluating water quality in aquaculture, as they accurately detect the presence of key compounds. This study relied on titrimetric methods to measure phosphates (PO_4_^3−^), alkalinity, total hardness, and the concentrations of calcium (Ca^2+^) and magnesium (Mg^2+^). These factors influence biological processes in fish ponds, and indicator values provide information on the nitrogen and phosphorus cycles, mineralization, and ionic balance. Titration procedures involved specific reactions and color-change indicators to determine equivalence points. Measurements of hardness, calcium, and magnesium were performed using complexometric titration with ethylenediaminetetraacetic acid (EDTA). Alkalinity was measured by titration with 0.1 N hydrochloric acid, and organic matter was measured based on chemical oxygen demand. These streamlined approaches aimed to accurately quantify key parameters and support the assessment of winter fish habitat quality.

*Digestion method*. First, about 15 mL of water was placed in a PM60 PTFE digestion tube. Next, 10 mL of digestion solution (7.5 mL HNO_3_, 2.5 mL HCl) was added under ambient conditions. The samples were allowed to digest statically for at least 2 h. After static digestion, the process continued in a TOPwave microwave system (Analytik Jena, Jena, Germany) in three steps: 145 °C for 5 min, 180 °C for 10 min, and 50 °C for 21 min, with a 5-min ramp for each step.

All chemicals used in this research were analytical grade (Merck KGaA, Darmstadt, Germany). To avoid spectral interference, dilutions were performed using Millipore Milli-Q ultrapure water (Merck KGaA, Darmstadt, Germany) with a resistivity of 18.2 MΩ·cm^−1^ at 25 °C.

### 2.3. Determination of Nitrites by Griess-Ilosvay Spectrophotometric Method

The Griess-Ilosvay spectrophotometric method is an analytical technique used to determine the concentration of nitrite ions (NO_2_^−^) in water samples [46,47]. This method is based on the diazotisation of an aromatic amine (e.g., sulfanilamide) with nitrite ions in an acidic medium, followed by the coupling of the resulting diazonium with another aromatic amine (α-naphthylamine), generating a colored azo compound (pink-red), whose absorbance is proportional to the concentration of NO_2_^−^ ions. Absorbance is measured at the optimal wavelength (520 nm) using an Evolution™ 260 Bio UV-Visible spectrophotometer (Thermo Fisher Scientific Inc., Waltham, MA, USA) previously calibrated with nitrite standard solutions. The high sensitivity of the method enables the determination of nitrite concentrations in the range of 0.01–1.00 mg/L [47], making it suitable for monitoring surface water bodies used for aquaculture, where even trace levels of nitrite can indicate malfunctions in nitrification processes and pose ecotoxicological risks. Briefly, the methods based on the Griess assay for determining nitrites in water samples from fishponds are outlined as follows: 50 mL of the water sample was mixed with 1 mL of 0.5% sulfanilic acid solution and 1 mL of 0.1% N-(1-naphthyl)-ethylenediamine solution. The mixture was left to stand for 15–20 min at room temperature (20–22 °C) in a light-protected environment to ensure the reaction was completed fully. Next, absorbance was measured spectrophotometrically at 520 nm, using a blank prepared with distilled water treated identically. NO_2_^−^ concentrations were determined by comparing the measured absorbances with a calibration curve created from standard solutions of sodium nitrite (NaNO_2_) over the range of 0.01–1 mg NO_2_^−^/L. The method’s relevance to analyzing water from fishponds is supported by the instability of nitrite in aquatic environments and its role as an indicator of recent nitrogen pollution or biological imbalances in the water column. Regular testing of NO_2_^−^ levels aids in water quality evaluation and provides a foundation for preventive measures in sustainable aquaculture management.

### 2.4. Microplastics Isolation Protocol

Water samples (8 L/sample) were collected in sterile glass containers cleaned with 70% nitric acid to minimize contamination [48]. All collection, handling, and filtration were performed in a controlled laboratory, following high hygiene standards per ISO 14644-1:2015 [49], with protective equipment (cotton lab coat, particle-free nitrile gloves) worn throughout. After sampling, 200 mL of 30% hydrogen peroxide (H_2_O_2_) was added to each sample to digest organic matter. Treated samples were held at room temperature (20–25 °C) for 24 h, shaken for 10 min, then left to stand until filtration. After chemical digestion, samples were filtered under vacuum using a three-position stainless steel filtration system (Labbox Labware, Barcelona, Spain), a vacuum pump (18 L/min), and 8–12 µm high-purity cellulose filters. Filters were transferred to pre-cleaned, sterilized glass Petri dishes for efficient particle retention.

### 2.5. Optical Microscopy

Optical microscopy (OM) was the first technique used to quantify and characterize the microparticles isolated from filters. The magnification levels range from 6.5× to 100×, depending on shape and size, using Primo Star and Stemi 2000-C microscopes (Carl Zeiss Microscopy GmbH, Jena, Germany) equipped with Axiocam 105 digital video camera (Carl Zeiss Microscopy GmbH, Jena, Germany) and ZEN 2012 (Version 1.1.2.0).

### 2.6. Micro-Fourier Transform Infrared Spectroscopy

One of the most widely used analytical methods for the chemical and morphological evaluation of microplastics is micro-Fourier Transform Infrared Spectroscopy (micro-FTIR). For this study, a Vertex 80v spectrometer coupled with a Hyperion 2000 IR microscope (Bruker Optics GmbH & Co. KG, Ettlingen, Germany) was used for chemical and morphological characterization of microplastics. The micro-FTIR system has the following characteristics: spectral range of 600–7500 cm^−1^, spectral resolution of 0.2 cm^−1^, and ±1 µm accuracy. The OPUS v.7.5 software was used to identify the polymer’s chemical structure based on the internal database.

### 2.7. Inductively Coupled Plasma Mass Spectrometry Analysis

Human health concerns involving fish as food require immediate and accurate investigation. It is noted that in trace element monitoring, measuring total toxicity often isn’t sufficient to ensure public safety. In this regard, inductively coupled plasma mass spectrometry (ICP-MS) is a reliable technique used for measuring trace metal concentrations (i.e., Cr, Fe, Ni, Co, Cu, Zn, Cd, and Pb) at large scales. The iCAP^TM^ Qc spectrometer (Thermo Fisher Scientific Inc., Waltham, MA, USA) is used for right-first-time analysis, with high accuracy and confidence in the quality of data from mineralized water samples. Before analysis, the ICP-MS system was carefully rinsed with a 5% HNO_3_ blank solution for 20 min. This removed any potential residuals from previous measurements. Each sample was analyzed in triplicate, and the average values were recorded as raw data; the analytical equipment was then rinsed for 2 min. Three replicate solutions were prepared for each experimental condition. Before ICP-MS analysis, all samples were treated with HNO_3_ until a pH of 1–2. Argon with a purity of 99.999% (Linde Gas, Bucharest, Romania) was used as the plasma, nebulization, and auxiliary gas in the ICP-MS analysis.

### 2.8. Analytical Quality Assurance

A series of QA/QC measures was implemented throughout the experimental procedure for both physicochemical analyses and the isolation of microplastics from the samples [50,51]. This ensured data reliability and minimised external or accidental contamination. All work steps were performed under the highest level of cleanliness. Work was performed in a sterile room to limit contamination from airborne microparticles. The glassware and filtration system were rigorously cleaned and rinsed with ultrapure water. This water was also filtered to avoid cross-contamination. Meanwhile, blanks were obtained by filtering a mixture of ultrapure water and hydrogen peroxide and then examining in similar conditions using both optical and micro-FTIR techniques; the results revealed no microplastic traces on the filter surface. Plastic materials were not used as blanks in the present study; based on the author’s experience, most microplastics in food and cosmetic samples were mixtures (i.e., natural and synthetic materials), not pure polymers [32,51]. All instruments and work surfaces were cleaned before use. Samples were handled using glassware. This helped avoid secondary contamination from airborne microparticles. All solutions (hydrogen peroxide, nitric acid, and distilled water) were filtered and capped when not in use. During filtration, the three positions of the filtration system were capped, except when adding water samples to the filtration system funnels [52,53]. Laboratory personnel wore cotton lab coats and limited movement during sample handling and filtration to reduce contamination with particles or fibres [54].

The concentrations of trace metals in acidified samples were assessed in triplicate using calibration curves derived from standard solutions analyzed by ICP-MS to ensure accuracy. Calibration was performed using the multi-element standard solution IV (Merck KGaA, Darmstadt, Germany), characterised by good linearity over the concentration range (i.e., 0.05 to 10.0 mg/L). All calibration curves exhibited R^2^ values of 0.999 or higher. An isotope with high abundance and minimal interference was chosen for each analysed element (i.e., Cd, Ni, Pb, Cr, Cu, Co, Zn, and Fe). To validate the method, the limit of detection (LOD), limit of quantification (LOQ), and recovery level (accuracy) were determined. Specifically, the LOD was calculated using the formula $\bar{B}+3\sigma$, while LOQ was calculated using the formula $\bar{B}+10\sigma$, where $\bar{B}$ represents the mean value in the blank solution, and σ is the standard deviation (Table 2). To evaluate the method’s accuracy, recovery tests were performed by spiking water samples with increasing amounts of each analyte at three levels (10, 20, and 40 ppb). The mean recoveries for the elements ranged from 80% to 120%.

### 2.9. Assessment of Pollution Level

The obtained values for metal content were used to assess the pollution level following the indices proposed by Banica et al. [59]:

Contamination factor (*CF_i_*):

(1) $${CF}_{i}=\frac{C_{i}}{QCI}-1$$

Contamination degree (*CD*):

(2) $$CD=\sum_{i=1}^{n}{CF}_{i}$$

Pollution load index (*PLI*):

(3) $$PLI=\sqrt[{n}]{\frac{C_{1}}{{QCI}_{1}}\times \frac{C_{2}}{{QCI}_{2}}\times \ldots \times \frac{C_{n}}{{QCI}_{n}}}$$

Heavy metal evaluation index (*HEI*):

(4) $$HEI=\sum_{i=1}^{n}\frac{C_{i}}{{QCI}_{i}}$$

Heavy metal pollution index (*HPI*):

(5) $$HPI=\frac{\sum_{i=1}^{n}W_{i}\times \frac{C_{i}}{{QCI}_{i}}\times 100}{\sum_{i=1}^{n}W_{i}}$$

where *C_i_* represents the concentration of element “i”; *QCI_i_* represents the value of element “i” for waters in first quality class (Romanian Order no. 161/2006 [55]); *W_i_* represents the unit weight of element “i” (the inverse value of *QCI_i_*): 0.040 for Cr, 0.003 for Fe, 0.100 for Ni and Co, 0.050 for Cu, 0.010 for Zn, 2.000 for Cd, and 0.200 for Pb) [59].

The HEI indicates the quality of a water sample, based on the following ranges: 0–50 (excellent quality), 51–100 (good quality), 101–200 (poor quality), and >201 (very poor quality) [59].

### 2.10. Statistical Analysis

Descriptive statistical analysis through IBM SPSS Statistics v. 27 (IBM, New York, NY, USA) was employed in this study to summarise the large dataset. Two main types of statistical analysis were used: data analysis and principal component analysis. Data analysis methods were used to obtain mean values and standard deviations, while PCA was used to correlate the water samples as a function of parameters and to identify similarities among the physicochemical data of fishpond water. Origin Pro 8.5 and Origin Pro 2019 (OriginLab Corporation, Northampton, MA, USA) were used to generate graphs.

## 3. Results and Discussion

Monitoring key parameters such as temperature, dissolved oxygen, pH, turbidity, salinity, nitrite, alkalinity, and water flow dynamics in relation to metal concentration is essential for assessing the balance of the rearing environment. Climate change directly influences the behaviour and balance of aquatic ecosystems, especially during winter. The formation of an ice layer can restrict gas exchange, leading to oxygen depletion and increased accumulation of toxic metabolites, such as ammonia and nitrite, due to limited biological filtration in winter. Additionally, low temperatures increase stress, further slowing fish metabolism and making them more sensitive to fluctuations in water quality parameters. The influence of precipitation, including rain and snowmelt, on the chemical balance of water is analyzed, with particular focus on dilution, nutrient leaching, and pH stability.

### 3.1. Trade-Offs Between Fishponds Water Quality, Sources, Nutrient Footprint or Supply, and Fish Growth in the Winter Season

The steady rise in average winter temperatures can disrupt or shorten fish hibernation, accelerate microbial activity, and alter oxygen solubility in water. These shifts can cumulatively degrade water quality, promoting physiological stress, unnecessary energy expenditure by fish, and increased risks of algal blooms and toxic build-up. Thus, air and water temperatures were monitored simultaneously during the period December 2024–February 2025, corresponding to the cold season (Table S1). Correlating these data allowed the assessment of the impact of climate variations on the thermal balance of fish ponds and on their capacity to support a favorable environment for species of interest in aquaculture. The statistical analysis highlighted direct relationships between decreases in air temperature and the thermal stabilization of water. These relationships directly affect critical factors, such as dissolved oxygen availability, metabolic rates in aquatic organisms, and the solubility of chemical substances, all of which are important for ecosystem stability and water quality management. Building on these findings, it also examined pH stability.

**Figure 2:**
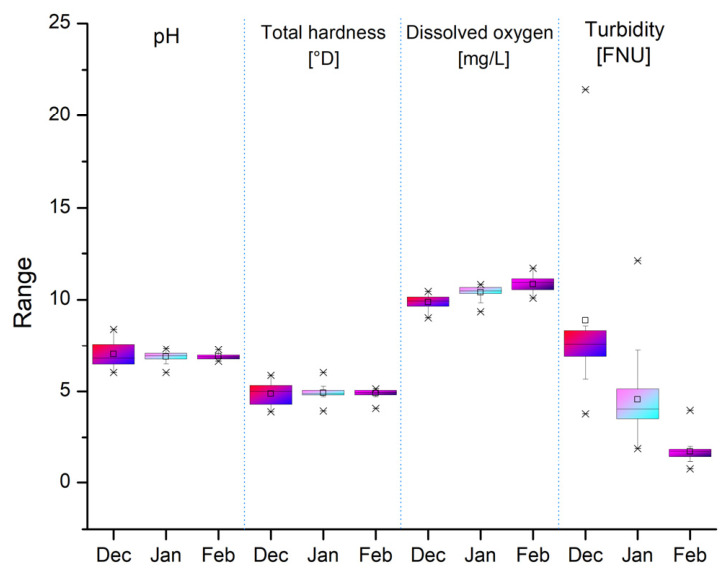
Monthly variation in pH, total hardness, dissolved oxygen, and turbidity in all analyzed water samples (fish ponds and Ilfov source): × marks the minimum and maximum values; □ marks the mean values; black line inside the box marks the median value.

The pH of the water remained relatively stable, ranging from 6.02 to 8.40, indicating slightly alkaline water. This stability is favorable for aquaculture fish according to general standards (optimal pH 6.5–8.5), supporting healthy habitat conditions (Figure 2). No significant variations were identified, suggesting that neither major external influences nor disruptive biological processes were present, thereby maintaining a suitable environment for aquaculture. The total hardness (expressed as °D, where 1 °D equals 10 mg/L of calcium oxide) ranged from 3.88 to 6.02 (Figure 2), corresponding to low hardness water that is suitable for most freshwater fish species, especially cyprinids. This indicates a balanced mineral salt content, particularly of calcium and magnesium, both essential minerals for aquatic life. An inverse correlation between temperature and DO was recorded. Dissolved oxygen levels remained within optimal parameters (>5 mg/L) throughout the winter, recording a maximum average of 11.71 mg/L (Figure 2), supported by a constant water supply and favorable water temperatures from December 2024 to February 2025.

Turbidity showed greater variability (0.74–21.43 FNU, where FNU stands for Formazin Nephelometric Units, a standard measure of water cloudiness), with notable maxima in basin BP6 and the minimum in SW (Figure 2). These increases may be correlated with local hydrodynamic disturbances, and the leaching of sediments following above-average precipitation in December 2024.

**Figure 3:**
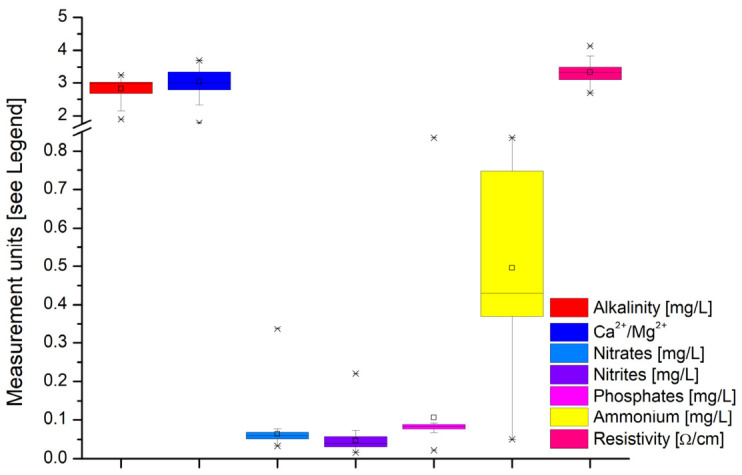
Variability of water quality parameters across all sampling sites: alkalinity (expressed as mg/L); Ca^2+^/Mg^2+^ (dimensionless); nitrate, nitrite, phosphate, and ammonium contents (expressed as mg/L); electrical resistivity (expressed as Ω/cm). The center line indicates the median value, the box represents the interquartile range (IQR), and the isolated dots (×) represent the outliers. × marks the minimum and maximum values; □ marks the mean values; black line inside the box marks the median value.

In terms of alkalinity, it was high (i.e., 1.90–3.25 mg/L, Figure 3), indicating a strong buffering capacity that contributes to pH stability and helps prevent acidification under thermal or biological stress. Calcium ions’ average concentrations were mostly constant (e.g., 18.80–27.70 mg/L), and magnesium ions ranged between 6.085 and 10.940 mg/L, with a Ca^2+^/Mg^2+^ ratio between 1.790 and 3.695 (Figure 3). The cold season is known to be a time when fish need particular care. These values suggest that the water should be balanced in terms of ions, to support the fish’s physiological development.

Nitrite and nitrite concentrations remained low across all analyzed water samples (i.e., 0.033–0.337 mg/L and 0.016–0.221 mg/L, respectively), as shown in Figure 3, reflecting the efficient functioning of nitrification processes, especially the activity of nitrite-oxidizing bacteria (*Nitrospira* and *Nitrobacter genera*), which convert nitrite to nitrate. Low NO_2_^−^ levels indicate advanced oxidation of nitrogen compounds and the stability of microbial communities involved in the nitrogen cycle, both of which are essential for maintaining water quality and preventing physiological stress on fish biota. Ammonium cations (NH_4_^+^) levels remained low (generally 0.050–0.835 mg/L), without dangerous accumulations (Figure 3); the reduced metabolism of fish in the cold season limits excretion, and nitrification remains functional. Phosphate concentrations were low (i.e., 0.022–0.835 mg/L, Figure 3), thereby reducing the risk of eutrophication in the cold season.

The electrical resistivity, ranging from 2.690 to 4.130 Ω/cm (Figure 3), i.e., with higher values indicating lower dissolved salt concentrations, confirms a moderate mineralization. This is directly correlated with the low water salinity (0.005–0.100‰), characteristic of freshwaters. These values suggest a constant supply of feedwater and a low biological activity, specific to the winter season.

**Figure 4:**
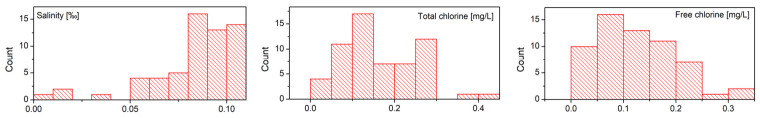
Frequency distribution of the average values determined for salinity (expressed as ‰), free and total chlorine content (expressed as mg/L). The count on the OY axis represents the number of calculated values in each range (monthly average values for each sampling area; the sum of counts per histogram is 60).

Regarding salinity, the lowest levels were observed in January and February 2025 (Figure 4). This followed heavier precipitation at the start of winter (i.e., November and December 2024). The lower average salinity values can be attributed to the influx of freshwater from early-season precipitation. The studied ponds host well-defined ecosystems with many common species (algae, diatoms, shells, etc.), likely because aquatic species adapt and compete effectively in freshwater environments. The artificial basins are also close together and experience minimal anthropogenic impact. The free and total chlorine contents showed minor differences between the water samples from the studied basins, with no significant deviations (i.e., 0.000–0.345 mg/L and 0.005–0.405 mg/L, respectively).

**Figure 5:**
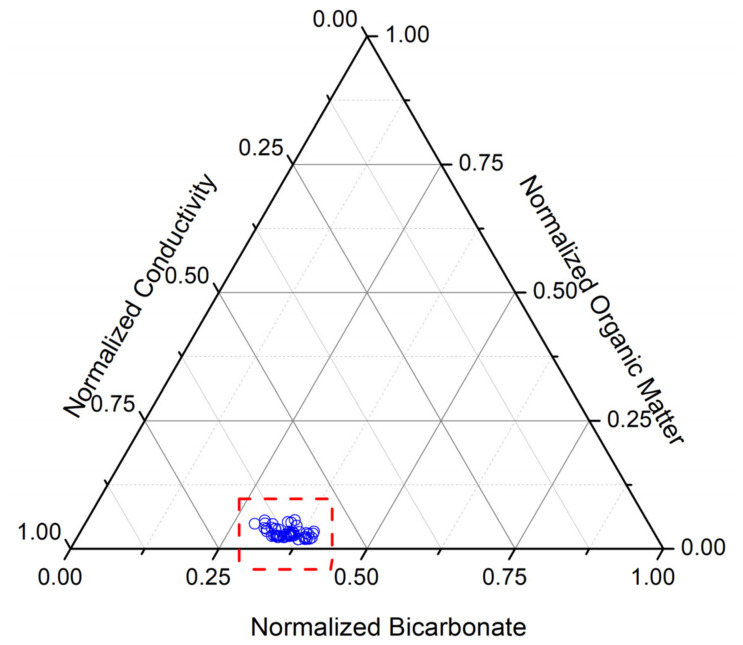
Ternary diagram of conductivity, organic matter, and bicarbonate; the normalized values were obtained using the equation: $z_{i}=\frac{x_{i}-min(x)}{\max\left(x\right)-min(x)}$ [60], to obtain a normalized range [0, 1]. All the analyzed basins (BPs) and the water source (SW) are marked with blue circles and are highlighted in the red rectangle.

The concentration of bicarbonate ions (115.90–198.25 mg/L) was consistent with the alkalinity values, indicating a stable anionic composition. The ternary diagram of conductivity, organic matter, and bicarbonate, based on the normalized values, is shown in Figure 5.

The chemical investigation of water samples showed that conductivity remained relatively constant throughout the cold season (early December 2024 through late January 2025). Average values ranged from 243.00 ± 43.98 to 345.50 ± 36.23 µS/cm. Values were slightly lower in January, likely due to above-average precipitation in December 2024. December temperatures were 2.5 °C above average, which resulted in a slight decrease in the concentration of dissociated salts in the basin water. These values are also explained by the lack of significant precipitation (below 5 mm) and by fewer frost and winter days. In January 2025, conditions were very warm (4.5 °C) and dry. Conversely, in February 2025, winter temperatures were normal, ranging from −12 °C to −2.5 °C, but precipitation was again very low, at below 5 mm (weather parameters are monitored daily by the SCDP Nucet). A comprehensive study related to the influence of weather factors on physicochemical parameters in SCDP Nucet presented data monitored in May–September 2022, compared to May–September 2023 [61]. The present study is complementary due to the monitored period (December 2024–February 2025).

The cold season 2024–2025 was characterized by physicochemical stability in all ponds (Table S2). This constancy is typical of the winter period, when biological processes are slow down, and external inputs are reduced. The parameters were in ranges favorable to fish health and aquatic ecological processes. However, this stability is increasingly challenged by climate change. Climate change, such as shifts in the precipitation regime, more frequent extreme events (torrential rains, persistent droughts from December 2024), and changes in snowmelt processes (i.e., lack of snow in January and February of 2025), disrupts the physicochemical stability observed in the ponds during the cold season by altering inflows and water quality, ultimately impacting the balance and health of aquatic ecosystems and implicitly of fish growth.

### 3.2. Effect of Metal Levels in Fishponds Water on Fish Growth in the Winter Season

**Figure 6:**
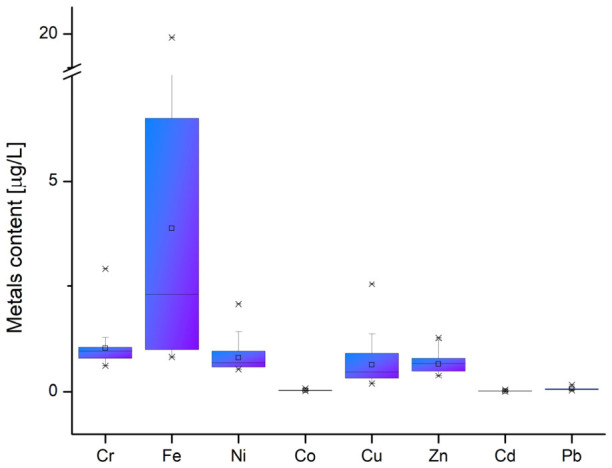
Box plots of metal concentrations (Cr, Fe, Ni, Co, Cu, Zn, and Pb) in water samples collected from 19 basins and the Ilfov River during December 2024–February 2026. All obtained values are lower than the values established for the first quality class by Romanian Order 161/2006 [55]. × marks the minimum and maximum values; □ marks the mean values; black line inside the box marks the median value.

Most metals play a dual role in the environment; depending on their concentration, they can be essential micronutrients or toxic to aquatic ecosystems [62]. Average metal contents in the fishpond water samples are listed in Figure 6. The average level of Pb in the fishpond waters ranged from 0.038 ± 0.020 (BP9) to 0.164 ± 0.060 (BP7) mg/L. Notably, the minimum value for Pb concentration in sample BP9 was measured in January 2025, while the maximum value in sample BP7 was found in December 2024. In fact, closer analysis revealed that lead concentrations in all water samples were higher in December 2024 than in January 2025. Lead can result from anthropogenic activities, including gasoline and tires, which release Pb residues into fishpond water.

Values obtained in February 2025 were higher than those in January 2025 but lower than those in December 2024. This trend was also observed for the other analyzed elements, including cadmium, chromium, nickel, cobalt, zinc, copper, and iron. Furthermore, it should be noted that the low cobalt concentrations in the analyzed samples clearly originate from the soil composition. Additionally, zinc mobilizes the mobile forms of cadmium. In nature, cadmium’s main sources are atmospheric deposition and the Earth’s crust. Cadmium is the most mobile metal and can readily move from soil to water and other media. Therefore, the presence of cadmium within permissible limits in the analyzed water samples is correlated with zinc’s soil mobility. On the other hand, chromium is a metal that cannot be degraded, so it still needs to be monitored; it primarily comes from vehicles and fertilizers. When ingested by biota, including fish, this metal accumulates in their tissues and poses a risk to humans.

Copper can originate from agricultural activities and be transported through the water supply. However, domestic and mechanical activities, as well as traffic, appear to contribute to increased copper emissions. The copper levels in the fishpond water samples were higher in December 2024 and were also associated with precipitation. Excessive copper levels are dangerous because they can be neurotoxic to fish.

Iron is essential for cellular life, but free iron can cause cellular damage and toxicity in fish. Iron as divalent ions (Fe^2+^), originating from sedimentary deposits, is present in freshwater in its reduced form (Fe^2+^), but upon contact with air, it oxidizes rapidly, converting to colloidal ferric hydroxide (Fe^3+^). The loss of dissolved carbon dioxide from the water contributes to the precipitation of iron; therefore, larger quantities are likely to be found in fishpond sediments. Iron oxidation also depends on pH; it occurs four times faster in alkaline conditions (pH 7.5–8.2) than in acidic ones (pH 6.02–6.50). In an aquatic medium, some metals are strongly adsorbed onto the mineral matrix or bound in stable complexes with organic matter or iron oxides, while others are highly mobile. It must be noted that metals such as cobalt, copper, zinc, and lead have a strong affinity for binding to iron oxides due to their high specific surface area and adsorption capacity. These oxides (i.e., sorbents) can act as natural “filters,” binding potentially toxic metals and thus reducing their bioavailability (e.g., through absorption by plants and organisms) in sediments [62]. The ability of iron oxides to immobilise these metals is central to understanding how their subsequent release, influenced by pH, affects environmental risk. In this respect, a neutral or weakly alkaline pH facilitates adsorption by deprotonating the iron oxide surface, increasing its negative charge by attracting metal cations. For instance, the interactions between cadmium and iron oxides, studied by Li et al., highlight how the oxidation process specifically affects the binding and release of cadmium in aquatic sediments [63]. Two other metals, i.e., Pb and Cu, exhibit a high affinity for iron oxides, forming very stable complexes that often bind more strongly than zinc or cadmium, according to a study conducted by Tamez et al. [64]. On the other hand, cobalt forms very stable complexes with iron oxides at higher pH [62]. In the monitored period, the presence of investigated metals is linked to climatic conditions of the cold season, particularly to December 2024 precipitation, which releases large quantities of metals from the atmosphere. Precipitation in the first winter month produced more acidic water (pH around 6.02–6.50). A slightly acidic pH correlates with higher iron concentrations, as iron is more soluble in acidic conditions. As pH rises toward alkaline levels, iron becomes less soluble and precipitates as insoluble hydroxides. The distribution of means for the analyzed metals in descending order was Fe > Cu > Ni > Zn > Cr > Pb > Cd > Co (Figure 6).

**Figure 7:**
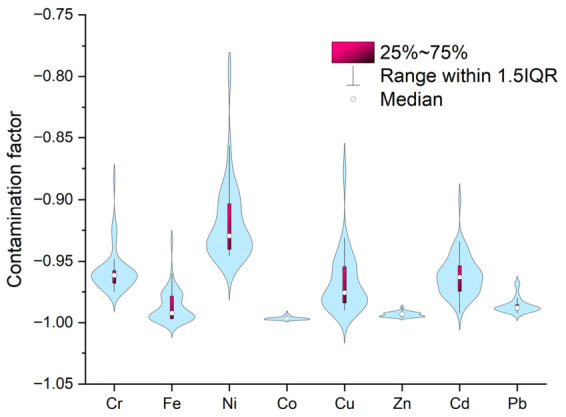
Violin graph of contamination factor calculated for all analyzed water samples: the pink bars represent 25–75% of the recorded values; the width of the violin represents the number of similar values, and the height is the difference between the maximum and minimum values; the IQR means interquartile range.

Based on the metal content data, the pollution indices were calculated. The contamination factor CF_i_ is an indicator used to assess the degree of pollution and anthropogenic impact on water bodies, and its ideal value is zero. In the current research, the order of metals in the surface water samples (in terms of average CF value) was Co (range −0.998–−0.992, average value −0.996) < Zn (range −0.996–−0.987, average value −0.993) < Fe (range −0.997–−0.934, average value −0.987) < Pb (range −0.992–−0.967, average value −0.986) < Cu (range −0.990–−0.873, average value −0.967) < Cd (range −0.992–−0.900, average value −0.962) < Cr (range −0.975–−0.883, average value −0.958) < Ni (range −0.945–−0.792, average value −0.918). Graphical representation of all data is shown in Figure 7.

According to Table 3, the obtained results highlight that all analyzed water samples are characterized by a low level of pollution. Similar results were obtained by Tyovenda et al. [65]: −0.987–−0.975 for Pb, −0.999–−0.995 for Ni, −0.999–−0.997 for Cu, −1.000–−0.985 for Cr, −0.9999–−0.9996 for Zn, and −0.998–−0.997 for Fe. Another similar study was led by Islam et al. [66] on groundwater samples with higher values than those obtained in the present study, characterized by a moderate level of pollution.

**Figure 8:**
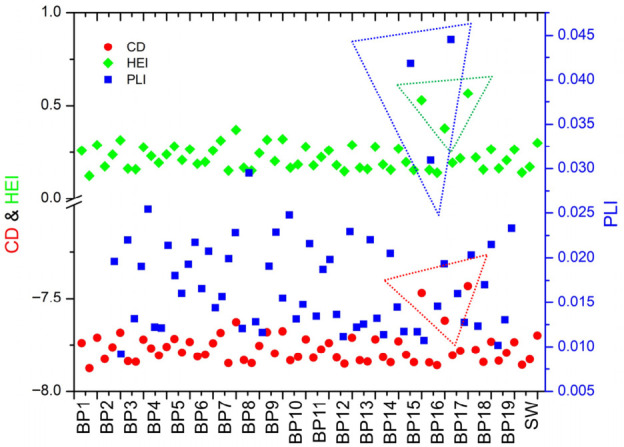
The obtained values for different pollution indices: CD—contamination degree; HEI—heavy metal evaluation index; PLI—pollution load index. Note: All parameters are dimensionless; dotted-line frames highlight samples with higher CD, HEI, and PLI values.

The violin plot represents the ideal graph for multiple data due to the fact that it uses the distribution of numerical data: the height represents the range of values, while the width represents data density at specific values (i.e., the wider sections indicate more data with similar values) [67]. The sum of CF for all determined metals represents the contamination degree—CD (Figure 8).

According to Table 3 and the CD results, all water samples are low-polluted (CD < 1). The same classification can be done using the HEI and PLI (Figure 8). It can easily be observed that the highest values were recorded for BP15, BP16, and BP17 in February 2025. Similar results for pollution load index were obtained by Tyovenda et al. [65], in the range of 0.020–0.030 (characterized by a low pollution degree), while higher values (PLI > 2) were reported by Islam et al. [66].

**Figure 9:**
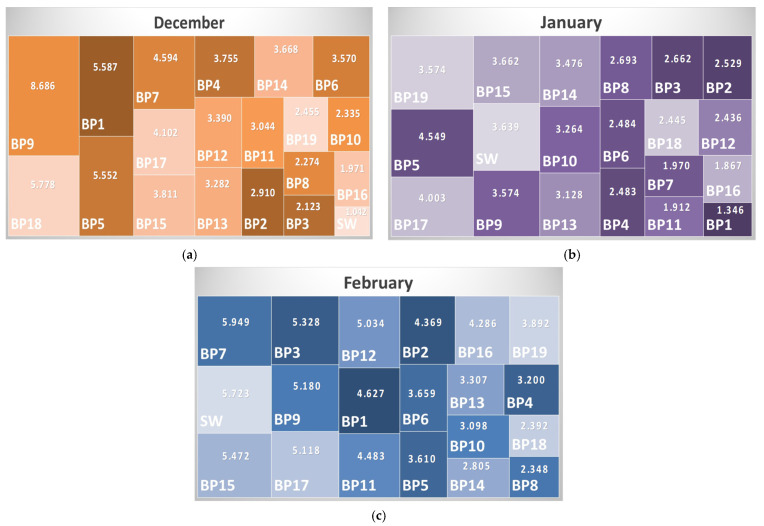
Hierarchy charts for the fishing ponds and Ilfov Stream based on HPI values for the monitored months: (**a**) December 2024; (**b**) January 2025; (**c**) February 2025. Note: The color shades do not mean higher or lower values.

Based on the data obtained for the HPI, the hierarchy charts were drawn (Figure 9).

The hierarchical structure of HPI is not similar for the monitored months, probably due to the differences related to the age and size of the fish in the pond, their number per pond, and the presence/absence of predators in the pond (species that reproduce during the cold period of the year).

### 3.3. Statistical Analysis of the Obtained Data

**Figure 10:**
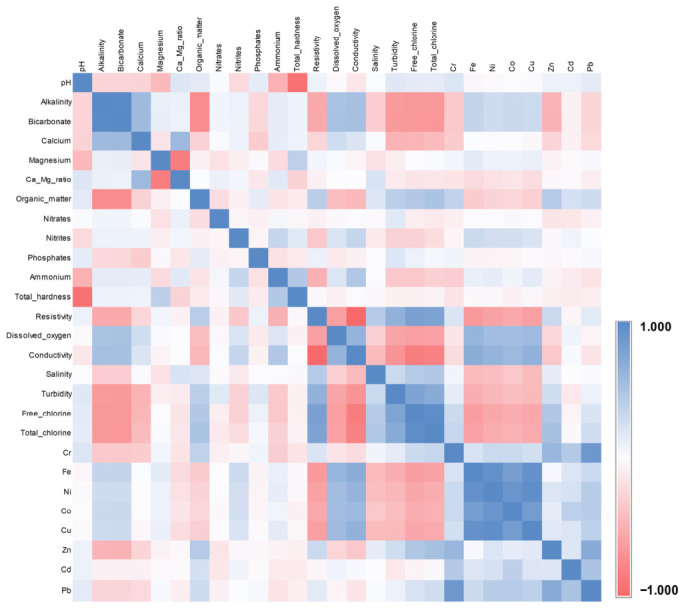
Graphical representation of the correlation coefficients between analyzed parameters (physicochemical indicators and metals): similarities between the data obtained in the fish ponds during the 3 months of monitoring.

The statistical analysis (principal component analysis) was used to generate the correlation matrix by SPSS Statistics software (v.27); these values highlight the similarities between different variables. In the present study, the similarities between all water samples and between all analyzed parameters (i.e., physicochemical indicators and metals) were presented as heat-maps (Figure S1 and Figure 10).

The correlation matrix presented in Figure 10 is characterized by a high similarity degree, even though some red spots are shown; even though the analyzed parameters have variable values, the basins are nearly identical. In a typical correlation matrix, green represents a perfect positive correlation (r = 1.000), while red/orange represents the “lowest” value in the set (r = 0.978). Therefore, Figure S1 suggests that the water in the analyzed basins (including Ilfov Stream) is a very stable environment, as confirmed also by the values of pollution indices.

The correlation matrix presented in Figure 10 shows the similarities between physicochemical parameters and the analyzed metals of all water samples. The correlation coefficients range from −1 to 1, highlighting strong positive and negative correlations as well as weak and medium correlations. Two strong positive correlations (r ≥ 0.700) observed in Figure 10 were recorded as follows: resistivity—free chlorine (r = 0.816), resistivity—total chlorine (r = 0.791); typically, between resistivity and chorines are negative correlations, but in this case, the recorded values are influenced by the self-purify process due to the partially stagnation in Ilfoveni Reservoir (Figure 1b). In a similar manner, turbidity has strong correlations with free chlorine (r = 0.783) and total chlorine (r = 0.719), explained by the abundant precipitations in the monitored period and the agricultural activities carried out upstream of SCDP Nucet. As it was expected, a strong correlation was recorded between iron and conductivity (r = 0.711), and it must be monitored because if this correlation coefficient increases, the dissolved oxygen decreases and affects the health and life conditions of fish [68].

Other strong positive correlations were observed between metals, as follows: Ni-Fe (r = 0.940), Co-Fe (r = 0.836), Co-Ni (r = 0.906), Cu-Fe (r = 0.940), Cu-Ni (r = 0.973), and Cu-Co (r = 0.852). All of these correlations are a witness to anthropic activities on the industrial platform of Targoviste City, Ilfov Stream passing near this area. The main sources of these toxic metals are represented by the production of stainless steel and other ferrous metals, where Ni is the main alloying element, and the wastewater discharges in which Fe and Ni concentrations increase during the acid pickling or washing steps of the technological process. In aqueous environments, metals can be influenced by pH variations, especially in acidic conditions, which favors the solubility of metals. It must be mentioned that the metal levels are low and the water quality is favorable for fish farming.

Another two strong positive correlations were observed in the pairs Pb-Cr (r = 0.883) and Pb-Zn (r = 0.726), explained by the industrial activities and particulate matter and smoke/tar generated by the furnaces washed by rainwater. High values of these three metals (i.e., Pb, Cr, and Zn) suggest a significant anthropogenic influence, a deficient pollution-control mechanism on the industrial platform, or that the Ilfov Stream receives highly polluted effluents [69]. The presence of Pb and Cr (simultaneously) increases the overall toxicity to fish, affecting the gills and liver more than if they were present individually [70].

Regarding the strong negative correlations, the following pairs must be mentioned: organic matter-alkalinity (r = −0.738), organic matter-bicarbonate (r = −0.738), total hardness-pH (r = −0.913), and conductivity-resistivity (r = −0.976). In the case of organic matter-alkalinity-bicarbonate, the negative correlations can be explained by the precipitation process of humic acids or the complexation process of organic matter with certain metals (especially Fe). The last group (i.e., conductivity-resistivity) represents a well-known and mandatory condition for all analyses, irrespective of the sample matrix.

The current investigation reveals the connection between the sources and environmental factors that influence the life cycles of fish species in the SCDP Nucet, as well as changes in water quality and nutrient supply. It provides a useful basis for further research into emerging contaminants, such as metals and microplastics. These contaminants could interfere with fish reproduction and growth. Therefore, integrated monitoring of all factors influencing fish development in artificial ponds is essential for optimizing growth conditions. This will contribute to the productive performance of aquaculture and the maintenance of a healthy, ecologically balanced environment.

### 3.4. Morphological and Chemical Characterization of Microplastics—Carriers for Metals: Risk to Pond Ecosystems

Microplastics, particles smaller than 5 mm, are a significant, often invisible, threat to water quality. With an estimated 51 trillion of these particles in the sea, their abundance far surpasses natural wonders like the stars in our galaxy [71]. The core concern is their potential impact on human health and the environment. While microplastics have been found in common foods (e.g., milk and dairy [32,72,73,74], salt [75,76], tea [77], juice [78], beer [79], honey [77], fish [80], fruits and vegetables [81], and water [82]), and the direct risks to humans remain uncertain, they may pose emerging dangers such as tumor formation, cognitive diseases [83], or malformations [84,85,86]. These risks are based on early scientific theories, not settled facts. Adding to the threat, microplastics can transport carcinogenic contaminants like metals [32,87], PAHs [32], viruses [88], and pesticides [89,90,91]. Microplastics act as vectors, intensifying the combined toxicity of the plastics themselves and the harmful substances they carry [92,93]. This synergy increases their impact on the environment and human health. The extent of these toxic effects depends on both the physical and chemical properties of the microplastics involved.

**Figure 11:**
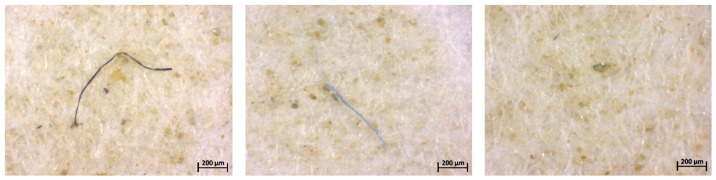

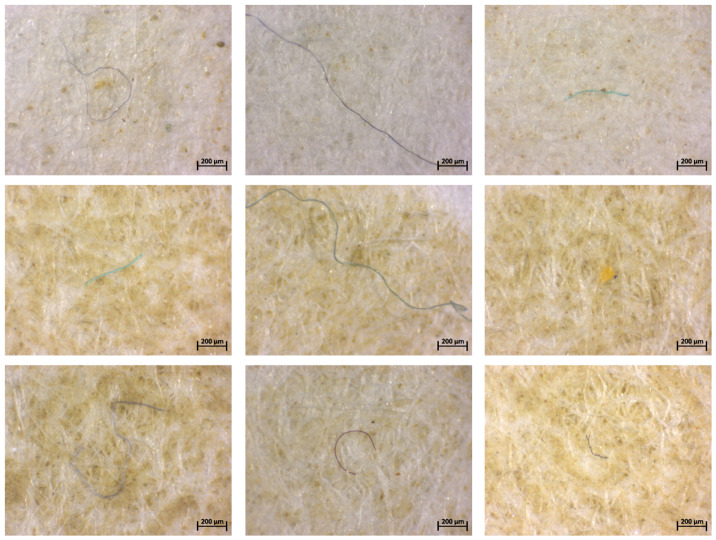
Optical microscopy images of representative microparticles identified on cellulosic filters resulted from the isolation process.

**Figure 12:**
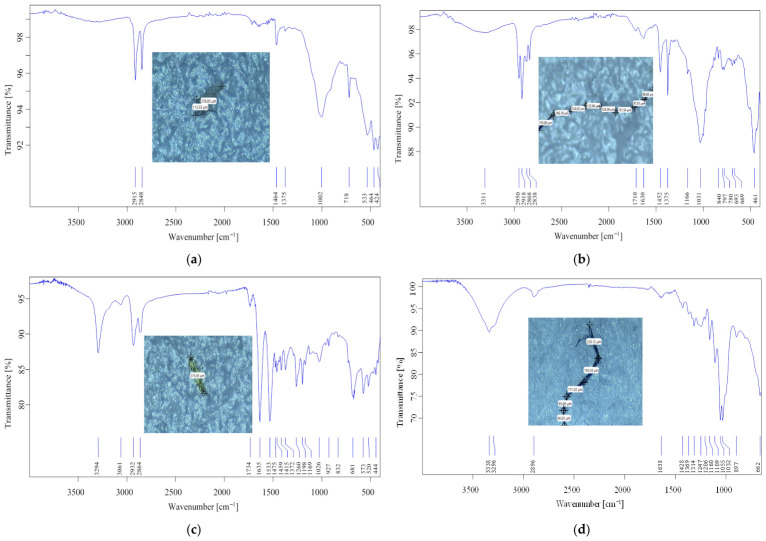
IR spectra of selected representative microplastics identified in surface waters: (**a**) polyethylene (PE); (**b**) polypropylene (PP); (**c**) polyamide (PA); (**d**) mixture cotton—poly(methyl)methacrylate (PMMA).

The first step was the quantification and chemical and morphological characterizations of microplastics in all collected water samples, including fishponds and the water source supplying the artificial ponds within SNCD Nucet, Romania. Obviously, the abundance of particles in open ponds increases directly with time of exposure to the environment, thereby increasing contamination. Given that December 2024 was a rainy month and January and February 2025 were lower in precipitation and unusually warm for the cold season, this can explain the number and diversity of microplastics in terms of color, shape, and size. Both optical microscopy and micro-FTIR spectroscopy provided information on the morphology and chemical structure of microplastics. Representative microplastics are shown in Figure 11 (optical microscopy images) and Figure 12 (IR spectra and measurement sequence).

Polyethylene, polypropylene, and nylon were among the most common polymer structures of microplastics found in the analyzed water samples. The ubiquity of these polymers is particularly concerning, given that they are used in the production of many consumer goods, such as sea salt and tea bags. Their low density (0.9–1.7 g/cm^3^) also makes them easily transportable in aquatic environments. Green, blue, and black microplastics in the form of fibers, as well as translucent or yellowish-white irregular fragments, were identified in the water samples analyzed, which were attributed to the polymers mentioned above. The most common microplastics found in the water samples after filtration is fiber type, and the most common polymers are polypropylene and polyethylene. The main sources of these anthropogenic particles are fishing nets, utensils, and plastic bags, as well as the residues from clothes/laundry [94]. Given the relatively even distribution of microplastics across the basins (between 10 and 20 microplastics/8 L of representative water sample), predominantly fibers, it can be concluded that their main source is daily anthropogenic activity by operators. Additionally, considering the microplastic cycle in nature, most often plastic fragments are carried by the wind into the freshwater of fish ponds. They may also be carried into the flowing surface water that feeds these ponds. Gradually, these fragments transform into thousands of microparticles. Fish often mistake these for their own food and ingest them. Thus, the presence of MPs in aquaculture systems poses a dual threat, endangering both the well-being of aquatic organisms and the safety and quality of aquaculture products. Microplastics in water can disrupt pond ecology, compromise fish physiology, and increase toxic exposure risks along the food chain.

In this regard, Zhang et al. found that microplastics can impair the intestinal structure of fish, known as shortened intestinal villi in Snakehead [95], disrupt immune responses, reduce growth, alter DNA, and heighten the absorption of other toxins such as *Oreochromis niloticus* [96]. In addition, Tang revealed that contamination with microplastics has been linked to neurotoxicity and quality degradation in aquaculture species [97].

Several authors have thoroughly studied the adsorption of metals onto the surface of microplastics [93,98,99,100]. These plastic particles adsorb cadmium, chromium, copper, iron, lead, and other metals due to their strong adsorption capacity. Notably, this capacity is, on the one hand, the result of the physicochemical properties of microplastics, including polymer type and color, functional groups, and, on the other hand, the water quality parameters such as pH, salinity, conductivity, dissolved oxygen, and organic matter [93,101]. Chen et al. reported that Cr(VI), a carcinogenic ion, is readily adsorbed onto aged microplastics due to changes in their adsorptive behavior [102]. However, microplastics bind to metals through electrostatic interactions, surface complexation, and non-specific interactions with metal-organic complexes.

Microplastics can adsorb various metal ions in aquatic environments, modifying their original toxicity. Aquatic animals often ingest microplastics because their color (blue, black, green, yellow), shape (fibers, fragments, films), and even size resemble their food. Microplastics that act as carriers of metals exhibit both synergistic and antagonistic effects on the entire aquatic ecosystem. For instance, Cao et al. revealed that the accumulation and persistence of both microplastics and metals in aquatic ecosystems pose challenges to amphibians due to the complex toxic effects [103].

On the other hand, microplastics with a high specific surface area, strong adsorption capacity, and the ability to undergo hard degradation can act as nutrient carriers for fish, providing a stable living environment and even a rich nutrient base [104]. In their research, Wootton et al. have shown that after 12 months of exposure, no clear signs of saturation points for metal content (i.e., Al, As, Ba, Co, Cu, Fe, Mn, Ni, and Pb) in LDPE [REF]; this observation indicates that microplastics may continue to adsorb metals over long time periods. At the same time, it is not necessarily that metals adsorbed on MPs are released in water, but after the biological processes, the toxic metals are released and accumulated in the fish meat (more specifically, *Hippocampus kuda* Bleeker) [105].

Microscopic plastic waste floating in the water attracts microorganisms such as bacteria, fungi, algae, and other single-celled organisms, which colonize the water, forming a unique microbial community in the fish’s common habitat.

To determine whether ingestion of microplastic-related metals increases chemical exposure risk in fish, additional long-term, environmentally relevant studies across various species are necessary. This need is underscored by the specific nature and pathways of microplastic-metal contamination in aquatic environments.

Furthermore, given the complex interactions outlined above, effective remediation strategies for metal ions adsorbed onto microplastics in natural aquatic systems remain underexplored. Additionally, the adsorption dynamics, combined with ecotoxicity and subsequent environmental risks, are not fully understood.

## 4. Conclusions

Farmed fish are much more sensitive to their environment because the water, which the fish depend on for oxygen and a number of other important chemicals, takes on a number of wastes that are not beneficial to aquatic life. Additionally, the survival of fish in ponds depends on water quality parameters, which are affected by climatic conditions, especially in winter. It is well known that winter-season physicochemical changes intensify contaminant adsorption and transformation, increasing bioaccumulation risk in fish and threatening the quality of the aquatic environment. To understand this impact, daily data on climatic and physicochemical parameters were collected over one winter (December 2024 to February 2025). Statistical analysis revealed the influence of winter weather on water quality, highlighting the links between air and water temperatures, physicochemical properties, contaminants, and the health of the aquatic ecosystem in fish ponds. Reduced levels of microplastics and metals were detected, indicating good water quality and an optimal aquatic system. Metals can be toxic in high concentrations and are adsorbed on microplastics, which would induce a potentially toxic effect on fish and aquatic organisms, as they are transported and accumulate through physical, chemical, and biological processes, damaging aquatic ecosystems. These findings directly support adaptive management strategies to maintain water quality and fish welfare during seasonal changes, emphasizing the need for timely action to address environmental threats. On the other hand, integrated monitoring of water parameters is vital for assessing diffuse pollution risks and guiding effective aquaculture management.

This particular scenario is the first step in a three-year research project focused on continuous monitoring of physicochemical parameters and contaminants, with a critical emphasis on the health of pond fish in a changing seasonal climate. To our knowledge, the key to minimizing the potential negative impact of climate conditions and contaminants on fish growth in ponds is to promote a wide range of innovative, adaptive technologies, such as Biofloc technology. This sustainable method will be implemented in Romania during a three-year project research, reducing the pollution load on receiving water resources through its no-discharge approach, eliminating additional water treatment costs through its biological cycles, and supporting the growth performance of raised animals, thereby reducing feed costs.

## Figures and Tables

**Table 1:** Fishponds monitored during the cold season.

No.	Sample Code	Main Characteristics of Fishponds: SA = Surface Area, Ht = Average Depth
1	BP 1	SA = 50,000 m^2^; Ht = 1.7 m
2	BP 2	SA = 10,140 m^2^; Ht = 1.6 m
3	BP 3	SA = 10,200 m^2^; Ht = 1.6 m
4	BP 4	SA = 3700 m^2^; Ht = 1.5 m
5	BP 5	SA = 4500 m^2^; Ht = 1.5 m
6	BP 6	SA = 2500 m^2^; Ht = 1.5 m
7	BP 7	SA = 2500 m^2^; Ht = 1.5 m
8	BP 8	SA = 2500 m^2^; Ht = 1.5 m
9	BP 9	SA = 5000 m^2^; Ht = 1.5 m
10	BP 10	SA = 20,200 m^2^; Ht = 1.6 m
11	BP 11	SA = 20,500 m^2^; Ht = 1.6 m
12	BP 12	SA = 1800 m^2^; Ht = 1.5 m
13	BP 13	SA = 2000 m^2^; Ht = 1.5 m
14	BP 14	SA = 2100 m^2^; Ht = 1.5 m
15	BP 15	SA = 1800 m^2^; Ht = 1.5 m
16	BP 16	SA = 2150 m^2^; Ht = 1.6 m
17	BP 17	SA = 2000 m^2^; Ht = 1.5 m
18	BP 18	SA = 2000 m^2^; Ht = 1.5 m
19	BP 19	SA = 2000 m^2^; Ht = 1.5 m
20	SW	Ilfov Stream: variable flow; Ht = 0.8 m

**Table 2:** Limits of detection (LOD) and limits of quantification (LOQ) for metals determined by iCAP Qc mass spectrometer in water samples. The standard values: national and proposed by the European regulatory bodies.

[μg/L]	Cr	Fe	Ni	Co	Cu	Zn	Cd	Pb
LOD	0.565	2.558	0.586	0.025	0.045	0.530	0.014	0.167
LOQ	1.081	6.304	1.390	0.054	0.095	1.172	0.034	0.417
Order 161/2006—First quality class (QCI) ^(1)^	25.000	300.000	10.000	10.000	20.000	100.000	0.500	5.000
Directive 2000/60/CE ^(2)^ amended by Directive 2013/39/UE ^(3)^	NA	NA	4.000	NA	NA	NA	0.080	1.200
Directive 2006/44/CE, cyprinid waters ^(4)^	NA	NA	NA	NA	40.000	1000.000	NA	NA

NA—Not Applicable. ^(1)^ Romanian Order No. 161 for the approval of the Norm regarding the classification of surface water quality to establish the ecological status of water bodies [55]. ^(2)^ Directive 2000/60/EC of the European Parliament and of the Council of 23 October 2000 establishing a framework for Community action in the field of water policy [56]. ^(3)^ Directive 2013/39/EU of the European Parliament and of the Council of 12 August 2013 amending Directives 2000/60/EC and 2008/105/EC as regards priority substances in the field of water policy [57]. ^(4)^ Directive 2006/44/EC of the European Parliament and of the Council of 6 September 2006 on the quality of fresh waters needing protection or improvement to support fish life [58].

**Table 3:** The levels of pollution as a function of indices value: *CF_i_*—contamination factor, *CD*—contamination degree, *PLI*—pollution load index, and *HEI*—heavy metal evaluation index [59].

Indices	Low Level of Pollution	Moderate Level of Pollution	High Level of Pollution
CF_i_	CF_i_ < 0	0 ≤ CF_i_ < 2	2 ≤ CF_i_
CD	CD < 1	1 ≤ CD < 3	3 ≤ CD
PLI	PLI < 2	2 ≤ PLI < 4	4 ≤ PLI
HEI	HEI < 40	40 ≤ HEI< 80	80 ≤ HEI

## Data Availability

The original contributions presented in this study are included in the article/Supplementary Materials. Further inquiries can be directed to the corresponding authors.

## Supplementary Materials

The following supporting information can be downloaded at: https://www.mdpi.com/article/10.3390/toxics14050403/s1, Figure S1: Heat-map in terms of similarities between all water samples and physicochemical indicators and metals; Table S1: Daily temperatures of water and air from December 2024 to February 2025; Table S2: Average values of chemical parameters in each fishpond during the monitored period (i.e., December 2024 to February 2026).
